# Supplementary material for: Angiotensin Receptor 1 Blockers Prolong Time to Recurrence after Radiofrequency Ablation in Hepatocellular Carcinoma patients: A Retrospective Study
Source: Biomedicines. 2020 Oct 8;8(10):399. doi: 10.3390/biomedicines8100399 (PMC7599746; doi:10.3390/biomedicines8100399)
Supplement: Supplementary file 1 [file biomedicines-08-00399-s001.pdf]

RFA

TTR	Recurrence	Last FU	Death	Therapy
3	0	18	1	n
3	1	18	1	n
3	1	21	1	n
3	1	21	1	n
4	1	49	1	ACE
6	1	8	1	n
6	1	8	1	n
6	1	21	1	n
6	1	21	1	n
6	1	88	1	ACE
8	1	10	1	n
8	1	18	1	n
8	1	18	1	n
8	1	24	1	n
8	1	49	1	n
8	1	49	1	n
9	1	11	1	n
9	1	111	1	ACE
9	1	11	1	n
9	0	11	1	n
11	1	14	1	n
11	1	14	1	ACE
12	1	21	1	n
12	1	21	1	n
12	1	34	1	n
12	0	34	1	ACE
12	1	58	1	n
12	1	58	1	n
14	1	20	1	ACE
14	0	20	1	n
14	1	25	1	n
15	1	25	1	n
15	1	25	1	n
15	1	31	1	n
15	1	31	1	n
15	1	91	1	sartan
15	0	31	1	n
15	1	65	1	n
18	1	24	1	n
20	1	35	1	sartan
23	1	42	1	n
23	1	42	1	n
24	1	51	1	n
24	1	71	1	n
26	0	29	1	n
26	1	29	1	n
26	1	29	1	n
26	1	29	1	n
27	1	42	1	n
31	1	36	1	ACE
31	1	35	1	sartan
31	1	70	1	ACE
31	1	70	1	n
32	1	78	1	n
33	1	48	1	n
33	0	48	1	ACE
34	1	55	1	sartan
35	1	160	0	n
21	1	37	0	n
36	1	48	1	sartan
36	1	62	1	n
15	1	28	0	n
38	1	42	1	n
38	1	59	1	n
42	0	48	1	n
42	1	48	1	sartan
42	1	49	1	n
42	1	51	1	n
42	1	51	1	n
42	1	58	1	sartan
42	1	98	0	n
44	1	56	1	sartan
44	1	85	0	ACE
44	0	85	0	ACE
46	1	68	1	n
46	1	47	1	sartan
47	1	51	1	ACE
47	1	54	1	n
48	1	49	1	sartan
19	1	31	1	sartan
53	1	87	1	ACE
11	0	22	1	sartan
56	1	88	1	ACE
58	1	69	1	n
58	1	69	1	ACE
58	1	91	0	sartan
15	1	25	1	sartan
64	1	72	1	ACE
14	0	34	0	ACE
64	1	88	0	n
69	1	84	1	sartan
72	1	79	1	sartan
72	1	79	1	n
72	1	89	1	ACE
72	0	89	1	ACE
72	1	75	1	ACE
77	1	92	1	sartan
77	1	92	1	sartan
2	0	27	1	n
3	0	95	0	n
3	0	95	1	n
5	0	24	1	n
5	1	28	1	n
5	1	28	1	n
6	1	48	1	n
6	0	48	1	n
8	0	72	1	ACE
8	0	72	1	n
18	0	18	1	n
18	0	18	1	ACE
38	0	38	1	n
38	0	38	1	ACE
41	1	41	1	n
41	1	41	1	ACE
46	1	46	1	n
58	0	58	1	n
70	0	70	0	ACE
72	0	72	0	ACE
72	0	72	0	n
73	0	73	1	sartan
74	1	74	0	ACE
74	0	74	1	ACE
75	1	75	1	sartan
77	1	77	0	n
77	0	77	1	sartan
77	1	77	0	ACE
77	1	77	0	n
77	0	77	0	n
78	0	78	0	n
78	0	78	0	ACE
78	1	78	0	n
78	1	78	0	ACE
78	0	78	0	n
80	1	80	0	ACE
82	0	82	1	n
82	0	102	1	ACE
83	0	103	1	sartan
86	1	86	1	n
86	1	188	1	ACE
88	1	188	0	ACE
88	0	188	0	sartan
88	0	88	0	n
88	1	188	1	sartan
89	1	89	0	n
90	0	90	1	sartan
98	0	98	1	ACE
99	0	99	1	n
18	1	108	0	n
188	1	208	0	sartan
122	1	188	0	n
166	0	166	0	sartan
166	0	166	0	n
166	0	166	0	sartan
3	1	21	1	n
3	1	21	1	n
3	1	21	1	n
3	1	21	1	n
3	1	21	1	n
3	1	21	1	n
9	1	11	1	ACE
9	1	11	1	ACE
9	1	11	1	ACE
9	1	11	1	ACE
9	1	11	1	ACE
9	1	11	1	ACE
9	1	11	1	ACE
9	1	11	1	ACE
9	1	11	1	ACE
15	0	151	1	sartan
15	1	15	1	sartan
150	1	75	1	sartan
15	0	51	1	sartan
15	1	15	1	sartan
15	1	15	1	sartan
15	0	65	1	sartan
15	1	65	1	sartan
33	1	48	1	n
33	0	48	1	n
33	1	48	1	n
33	1	48	1	n
33	0	48	1	n
33	1	48	1	n
14	1	34	0	ACE
14	1	34	0	ACE
14	1	14	1	ACE
14	1	14	1	ACE
14	1	104	1	ACE
14	1	14	1	ACE
14	1	68	0	ACE
33	0	33	0	sartan
33	0	65	0	sartan
33	0	73	1	sartan
33	0	33	1	sartan
33	0	33	1	sartan
33	1	33	0	sartan
33	0	33	1	sartan
33	1	42	1	ACE
23	1	42	1	ACE
23	0	42	1	ACE
23	1	42	1	ACE
23	1	42	1	ACE
23	0	42	1	ACE
23	1	42	1	ACE
33	0	48	1	n
33	1	48	1	n
33	1	48	1	n
33	1	48	1	n
33	1	48	1	n
33	0	48	1	n
33	1	48	1	n
33	0	48	1	n
33	1	48	1	n
33	1	48	1	n
33	0	48	1	n
33	1	48	1	n
